# Anti-Idiotypic Antibody Specific to GAD65 Autoantibody Prevents Type 1 Diabetes in the NOD Mouse

**DOI:** 10.1371/journal.pone.0032515

**Published:** 2012-02-24

**Authors:** Xin Wang, Aixia Zhang, Yu Liu, Shi Chen, Zhenqing Feng, Wenbin Shang, Marlena Maziarz, Jared Radtke, Christiane S. Hampe

**Affiliations:** 1 Department of Endocrinology, Jiangsu Province Hospital of TCM, Nanjing, Jiangsu, China; 2 School of Pharmacy, Nanjing Medical University, Nanjing, Jiangsu, China; 3 Department of Endocrinology, 2nd Hospital of Jilin University, Changchun, Jilin, China; 4 Key Laboratory of Antibody Technology of Ministry of Health, Nanjing Medical University, Nanjing, Jiangsu, China; 5 Medical Research Center, First College of Clinical Medicine, Nanjing University of Chinese Medicine, Nanjing, Jiangsu, China; 6 University of Washington, Seattle, Washington, United States of America; Weizmann Institute of Science, Israel

## Abstract

Overt autoantibodies to the smaller isoform of glutamate decarboxylase (GAD65Ab) are a characteristic in patients with Type 1 diabetes (T1D). Anti-idiotypic antibodies (anti-Id) directed to GAD65Ab effectively prevent the binding of GAD65 to GAD65Ab in healthy individuals. Levels of GAD65Ab-specific anti-Id are significantly lower in patients with T1D, leading to overt GAD65Ab in these patients. To determine the possible protective role of GAD65Ab-specific anti-Id in T1D pathogenesis, we developed the monoclonal anti-Id MAb 8E6G4 specifically targeting human monoclonal GAD65Ab b96.11. MAb 8E6G4 was demonstrated as a specific anti-Id directed to the antigen binding site of b96.11. MAb 8E6G4 recognized human antibodies in sera from healthy individuals, T2D patients, and T1D patients as established by ELISA. We confirmed these MAb 8E6G4-bound human antibodies to contain GAD65Ab by testing the eluted antibodies for binding to GAD65 in radioligand binding assays. These findings confirm that GAD65Ab are present in sera of individuals, who test GAD65Ab-negative in conventional detection assays. To test our hypothesis that GAD65Ab-specific anti-Id have an immune modulatory role in T1D, we injected young Non Obese Diabetic (NOD) mice with MAb 8E6G4. The animals were carefully monitored for development of T1D for 40 weeks. Infiltration of pancreatic islets by mononuclear cells (insulitis) was determined to establish the extent of an autoimmune attack on the pancreatic islets. Administration of MAb 8E6G4 significantly reduced the cumulative incidence rate of T1D and delayed the time of onset. Insulitis was significantly less severe in animals that received MAb 8E6G4 as compared to control animals. These results support our hypothesis that anti-Id specific to GAD65Ab have a protective role in T1D.

## Introduction

Autoantibodies to the 65 kd isoform of glutamate decarboxylase (GAD65Ab) are well-recognized humoral markers of the autoimmune response of type 1 diabetes (T1D) [1]. However, our recent findings suggest that GAD65Ab exist also in healthy individuals, where their binding to GAD65 is blocked by specific anti-idiotypic antibodies (anti-Id) [2]. The serum concentration of GAD65Ab-specific anti-Id in T1D patients is significantly lower as compared to that in healthy individuals, resulting in the easy detection of GAD65Ab using conventional radioligand binding assays [2].

According to the “network hypothesis” [3], anti-Id and autoantibodies co-exist to maintain the homeostasis of the immune system. An imbalance of this network may induce autoimmune diseases and a negative correlation between anti-Id and autoimmune disease has been demonstrated in autoimmune diseases, such as systemic lupus erythematosus (SLE), Hashimoto's thyroiditis, Graves' disease, Myasthenia Gravis, and Sjögren's syndrome [4]–[10]. Naturally occurring autoantibody-specific anti-Id can be detected in relatives of SLE patients [11], individuals who were in contact with SLE patients [12], and even in healthy controls [7], [13], [14]. In marked contrast, these anti-Id are not present in most patients with active SLE [15], [16]. However, patients in remission from SLE show a resurgence of anti-Id [7], suggesting a protective role of anti-Id.

Similarly, anti-Id specific to autoantibodies characteristic for Graves' disease are associated with remission in Graves' disease [17], and have been linked to a better response of patients to anti-thyroid drugs [18]. These findings further support the notion that autoantibodies are present in healthy individuals but concealed by the presence of anti-Id.

The role of anti-Id in the development of autoimmune diseases is unclear. A potential regulatory function of anti-Id is through neutralization of pathogenic autoantibodies, a mechanism that may explain the beneficial use of Intravenous Immunoglobulin in treatment of autoimmune diseases [19].

Previously, we demonstrated that injection of the T1D-associated human monoclonal GAD65Ab b96.11 into young non-obese diabetic (NOD) mice induced b96.11-specific anti-Id and significantly reduced the morbidity of T1D in the animals [20]. To elucidate the role of anti-Id in the regulation of the autoimmunity response, we developed a monoclonal anti-Id targeting the antigen binding site of b96.11. This murine anti-Id (MAb 8E6G4) is b96.11-specific and targets the antigen binding site of b96.11, preventing binding of GAD65 to b96.11 and not to other GAD65Ab. MAb 8E6G4 was successfully used to detect GAD65Ab in sera of individuals, who tested GAD65Ab-negative in conventional detection assays. We found that the levels of masked GAD65Ab in T1D patients and T2D patients were significantly higher than those in healthy individuals. Finally, injection of NOD mice with MAb 8E6G4 significantly reduced severity of insulitis and resulted in reduced incidence rate of diabetes. These findings suggest an immune modulatory role of GAD65Ab-specific anti-Id in the development of T1D.

## Results

### Development of monoclonal antibody 8E6G4 specific to b96.11

Animals responding to b96.11-injections with production of b96.11-reactive antibodies were identified by ELISA (data not shown). One animal showed good binding to b96.11 even at 1∶256,000 dilution, while no binding to the control antibody hLF was detected. Hybridoma 8E6G4 was generated from this animal.

### MAb 8E6G4 binds to human GAD65Ab b96.11 with high specificity and affinity

**Figure 1:**
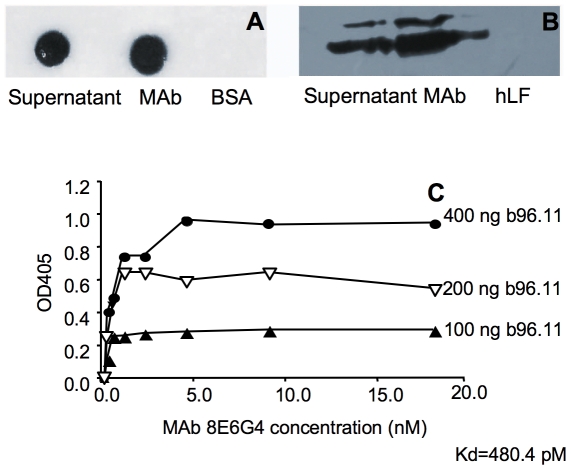
MAb 8E6G4 binds specifically to b96.11. **A:** Purified MAb 8E6G4, supernatant of hybridoma 8E6G4, or BSA were analyzed for binding by b96.11 by Dot blot. From left: supernatant of hybridoma 8E6G4, purified MAb 8E6G4, and BSA. **B:** Binding of MAb 8E6G4 and supernatant of hybridoma 8E6G4 to b96.11 was analyzed by immunoprecipitation followed by Western blot analysis. Binding of MAb 8E6G4 to control antibody hLF served as negative control. From left: supernatant of hybridoma 8E6G4 with b96.11, MAb 8E6G4 with b96.11, and MAb 8E6G4 with hLF. **C:** MAb 8E6G4 binds to b96.11 with high affinity. Dissociation constant was calculated by non-competing ELISA. Different concentrations of b96.11 were tested for binding by MAb 8E6G4. Kd of each concentration was calculated. The Kd was calculated as follows: Kd = 2(nKd′-Kd)/(n-1), where n = [b96.11]′/[b96.11]. The average Kd calculated was 480.4 pM.

We characterized the binding of MAb 8E6G4 to b96.11 by dot-blot, ELISA, and immunoprecipitation. The dot blot analysis demonstrated that b96.11 showed significant binding to MAb 8E6G4 (both purified and supernatant) and did not bind BSA (Figure 1A). This specificity of MAb 8E6G4 was further established in immunoprecipitations where MAb 8E6G4 was incubated with b96.11 or control antibody hLF. The immune complexes were immunoprecipitated with Protein L-agarose [21], which binds only to kappa light chains and not lambda light chains. As b96.11 contains a lambda light chain this allowed the specific precipitation of MAb 8E6G4. Only b96.11 and not the control antibody hLF were detected in the subsequent analysis of the co-immunoprecipitated antibody by Western Blot analysis (Figure 1B). The dissociation constant (Kds) of MAb 8E6G4 to b96.11 was determined as 480.4 pM by non-competing ELISA (Figure 1C).

### MAb 8E6G4 binding to GAD65Ab b96.11 is specifically blocked by GAD65

**Figure 2:**
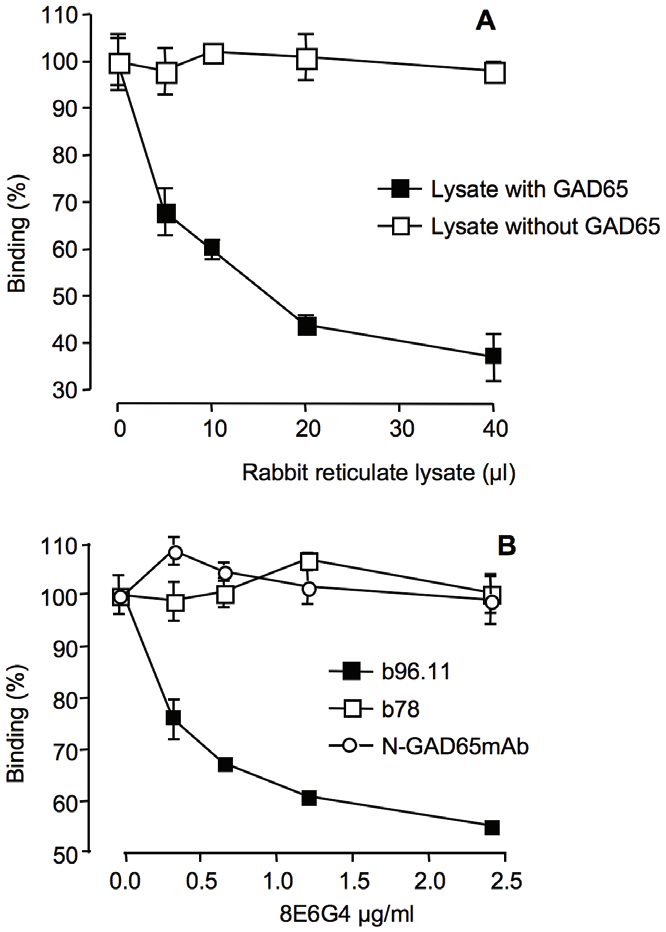
MAb 8E6G4 competes with b96.11 for binding to GAD65. **A:** GAD65 was translated *in vitro* using rabbit reticulocyte lysate. Different amounts (0–40 µl, corresponding to 0–160 ng) of recombinant GAD65 were incubated with 1.4 µg/ml MAb 8E6G4 and subsequently analyzed for binding to b96.11 by ELISA. Rabbit reticulocyte lysate alone was used as a negative control (open squares). Binding in the absence of competitor was set as 100%. **B:** Binding of monoclonal GAD65Ab b96.11 (black squares), b78 (open squares), and N-GAD65mAb (open circles) to radiolabeled GAD65 in the presence of the indicated concentrations of MAb 8E6G4 was determined. Binding is reported at percent binding, binding of the respective antibody to radiolabeled GAD65 in the absence of MAb 8E6G4 is set as 100%.

To establish the binding site of MAb 8E6G4, binding of MAb 8E6G4 to b96.11 was competed with recombinant GAD65 in a competitive ELISA (Figure 2A). We found that recombinant GAD65 significantly reduced MAb 8E6G4 binding to b96.11 in a specific and dose-dependent manner. This result strongly indicated that MAb 8E6G4 recognizes the antigen binding site of b96.11.

### Binding of MAb 8E6G4 to GAD65Ab b96.11 is antibody-specific

The epitope specificity of MAb 8E6G4 was further explored by testing the binding of MAb 8E6G4 to b96.11 and other monoclonal GAD65Ab. Human GAD65-specific monoclonal antibodies b96.11 and b78 and mouse GAD65-specific monoclonal antibody N-GAD65mAb were incubated at their respective half-maximal binding concentrations (150, 800, and 40 ng/ml, respectively) with different concentrations of MAb 8E6G4 (0–0.32 µg/ml). Binding of the GAD65-specific monoclonal antibodies to radiolabeled GAD65 in the presence of MAb 8E6G4 was analyzed by RBA (Figure 2B). We found that only binding of b96.11 to GAD65 was inhibited by MAb 8E6G4, while binding of GAD65 by b78 or N-GAD65mAb was not affected. These findings support that MAb 8E6G4 is a b96.11-specific anti-Id that binds to the antigen-binding region of b96.11.

### Sequence analysis of the heavy and light chain of MAb 8E6G4

The variable region protein sequences deduced from the nucleotide sequences were aligned for comparison with homologous sequences from the NCBI data bank. The nucleotide sequences for both heavy and kappa chain genes were submitted to GenBank under accession numbers JF345172 and JF345173, respectively. Sequence analysis of the heavy-chain-specific cDNA revealed that the heavy chain belonged to the mouse IgG heavy chain subgroup 1. Sequence analysis of the light-chain cDNA and database comparison revealed that the light belonged to the mouse kappa light-chain group 4–59. Comparison of the nucleotide sequences of the heavy- and light-chains with GenBank databases revealed no identity to previously reported sequences. The accuracy of the deduced amino acid sequence was verified by Mass Spectrometry analysis of purified MAb 8E6G4.

### GAD65Ab in human sera bind to MAb 8E6G4

**Figure 3:**
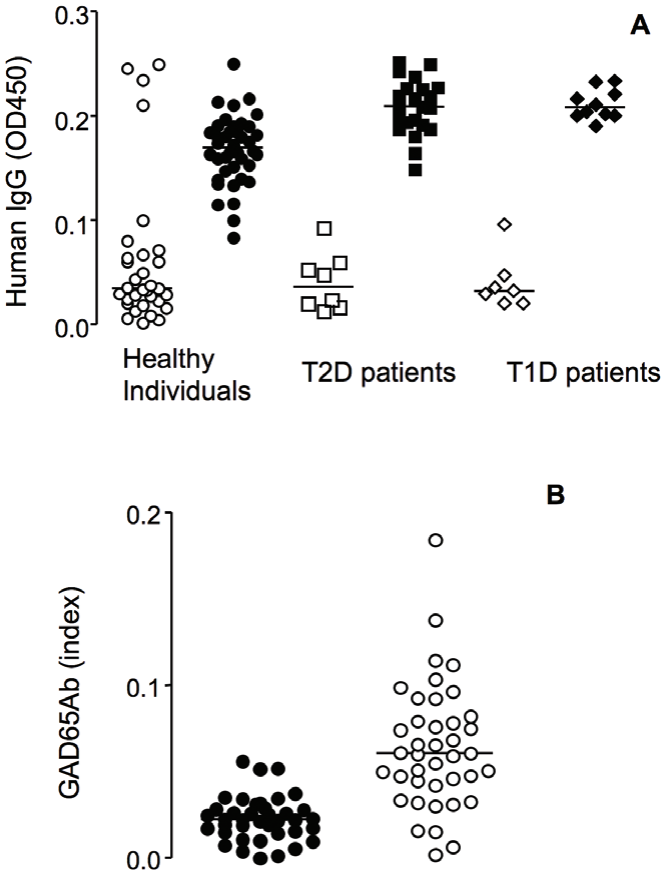
MAb 8E6G4 binds GAD65Ab present in human sera. **A:** Human sera obtained from GAD65Ab-negative healthy individuals (circles) (n = 40), GAD65Ab-negative T2D patients (squares) (n = 22), and T1D patients (diamonds) (n = 10), were analyzed for binding to MAb 8E6G4 by ELISA. Binding of human sera to three mouse monoclonal antibodies specific to human HGF is shown as a negative control (open symbols). Median binding is indicated. **B:** Human sera obtained from healthy individuals (n = 40) (GAD65Ab-negative in RBA) were tested for presence of masked GAD65Ab. Sera were precipitated on immobilized MAb 8E6G4, and subsequently eluted. Elutions were tested for presence of GAD65Ab by RBA. GAD65Ab titers of sera prior to precipitation (black circles) and elutions (open circles) are presented as GAD65Ab index. Median binding is indicated.

Previously we reported that sera that tested GAD65Ab-negative in conventional RBA, contained GAD65Ab in complex with anti-Id [2]. Removal of anti-Id allowed the detection of the GAD65Ab. To confirm these earlier findings we tested whether MAb 8E6G4 could detect GAD65Ab in sera of individuals, especially in healthy individuals and T2D patients, whose sera did not contain overt GAD65Ab. Initially we tested sera obtained from GAD65Ab-negative T2D patients (n = 22), healthy individuals (n = 40), and T1D patients (n = 10) by ELISA (Figure 3A). Of the ten T1D patients six sera tested GAD65Ab-positive.

We observed that human antibody bound specifically to MAb 8E6G4, while only four of the T2D patients' sera showed binding to the control mouse antibodies. Binding levels in T1D patients and T2D patients significantly exceeded those observed in healthy individuals (p<0.0001). Binding levels in T1D patients and T2D patients did not differ significantly. Human antibodies in sera from GAD65Ab-positive and GAD65Ab-negative T1D patients bound equally well to MAb 8E6G4.

We established that the MAb 8E6G4-bound human antibody contained GAD65Ab by testing the eluted antibody for binding to GAD65 (Figure 3B). Sera of 40 GAD65Ab-negative healthy individuals were incubated with MAb 8E6G4-PAS and subsequently eluted with high pH. Elutions were immediately neutralized and tested for binding to GAD65 in RBA. Binding levels significantly increased (from a median GAD65Ab index of 0.02 to a median GAD65Ab index of 0.06) (p<0.0001). No significant increase in binding to islet cell autoantigen IA-2 was observed (data not shown). These findings confirm our earlier observation of GAD65Ab in sera of healthy individuals.

### Injections of NOD mice with MAb 8E6G4 significantly delays the onset and reduces the incidence rate of diabetes

**Figure 4:**
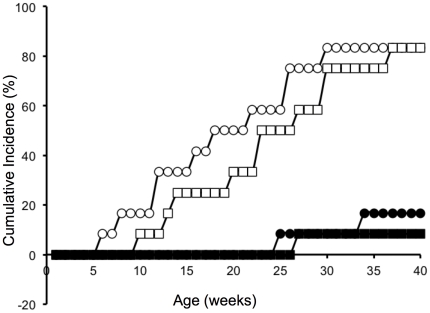
Cumulative incidence rate of diabetes development. NOD mice were injected with 50 and 100 µg MAb 8E6G4, presented as black circles and squares, respectively. Control animals were injected with PBS (white circles) or control mouse monoclonal antibody (white squares). Animals were weekly monitored for diabetes development. Cumulative incidence rate of diabetes development is presented as percentage.

Animals were injected with MAb 8E6G4, a control mouse monoclonal antibody, or PBS. The animals were monitored weekly for diabetes development. Mice injected with PBS started to develop diabetes at five weeks of age and 10/12 (83%) animals developed diabetes by week 30, confirming the expected natural development of diabetes in NOD mice. Injection with control monoclonal antibody had no significant effect on the development of T1D. However, injections with 50 and 100 µg MAb 8E6G4 yielded a cumulative incidence rate of 8%, and 17%, respectively. The animals in the lower dose group developed diabetes at week 25 and 32, the only animal in the 100 µg group developed diabetes at week 27 (Figure 4). The reductions in incidence rate and delay in onset observed in animals injected with 50 µg or 100 µg MAb 8E6G4 was significant compared to control animals (p = 0.0006 and p = 0.0002, respectively).

### MAb 8E6G4 reduces the severity of insulitis

**Figure 5:**
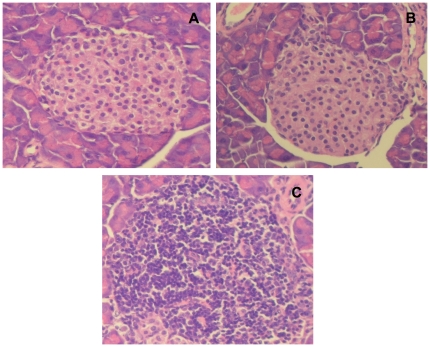
The histopathology of pancreatic islets at onset of diabetes or at 40 weeks of age. Representative images of pancreatic islets from animals injected with 50 µg (A) and 100 µg (B) MAb 8E6G4 IgG, or control animals (C). Pancreatic tissues were sectioned and stained with hematoxylin and eosin.

The above effect was also reflected in the severity of insulitis. Insulitis was less severe in animals treated with 50 and 100 µg MAb 8E6G4 IgG as compared to the animals injected with PBS or control IgG (Table 1, Figure 5).

**Table 1:** Degree of insulitis in NOD mice.

Score	1	2	3	4	5
**MAb 8E6G4 50 µg**	58	37	5	0	0
**MAb 8E6G4 100 µg**	60	34	0	2	0
**PBS**	0	0	13	17	70
**Control IgG**	0	0	12	17	71

Islets obtained from animals injected with MAb 8E6G4 IgG (50 and 100 µg), control IgG, and PBS were scored as normal islets (score 1), perivascular/periductal infiltration (score 2), peri-insulitis (score 3), mild insulitis (<25% of the islet infiltrated; score 4), and severe insulitis (more than 25% of the islet infiltrated, score 5). The mean score for each group was calculated by dividing the total score by the number of islets scored.

## Discussion

In this study, we tested our hypothesis that anti-Id specific to GAD65Ab have a protective immune modulatory role in the development of T1D. We developed and characterized a monoclonal anti-Id (MAb 8E6G4) targeting the antigen-binding site of human monoclonal GAD65Ab b96.11. This murine antibody is specific to b96.11 and blocks binding of GAD65 to b96.11 meeting the criteria of a b96.11-specific anti-Id. MAb 8E6G4 bound to both overt and masked human antibodies in sera of T2D patients, T1D patients, and healthy individuals. We showed that the MAb 8E6G4-bound human antibodies contained GAD65Ab, confirming our earlier findings of the presence of GAD65Ab in healthy individuals [2]. The significantly higher levels of masked GAD65Ab in T2D patients as compared to healthy individuals may suggest islet autoreactivity in these patients [22]. Follow-up studies to determine the development of autoimmune diabetes in these patients are necessary to confirm this hypothesis.

Administration of MAb 8E6G4 to prediabetic NOD mice successfully reduced the severity of insulitis in the animals. Consequently, diabetes incidence was significantly reduced and age of onset was delayed as compared to control animals. This effect was antibody-specific, since administration of control mouse monoclonal antibody had no effect on the development of diabetes.

Successful prevention of diabetes in NOD mice has been achieved by a variety of approaches, including vaccination with autoantigens, manipulation of immune cells, cytokine or vitamin treatment [23]–[26]. While the isotype-control in the NOD injection protocol suggests that the preventative effect of MAb 8E6G4 is antibody specific, more studies are necessary to confirm this anti-Id as a novel treatment approach in NOD mice. In future studies we will determine the mechanisms by which MAb 8E6G4 affects the immune response. MAb 8E6G4 may function as an internal image of the antigen-binding site of b96.11, thereby mimicking GAD65 vaccination. Alternatively MAb 8E6G4 could modulate antigen presentation as discussed later.

In the Idiotypic Network Hypothesis, Jerne postulated that under normal condition antibodies are kept in homeostasis by a network of idiotypic antibodies (Ab1) and their anti-Id (Ab2) [3]). When the anti-Id binds to the complementary determine region (CDR) of Ab1, it can compete the binding of Ab1 to antigen [27], [28]. The invasion of pathogen or the exposure of autoantigen breaks the balance and leads to the domination of Ab1.

T1D is recognized as a cell-mediated autoimmune disease [29]. However, recent studies indicate a role of the humoral immune response in the pathogenesis of T1D, mainly through modulation and/or mediation of antigen presentation. Autoantibodies can enhance antigen presentation, thus lowering the antigen concentration necessary for T cell stimulation [30]. Depletion of B lymphocytes by anti-CD20 antibody delays onset of T1D in NOD mice [31], and T1D patients treated with anti-CD20 showed improved islet beta-cell function, indicating an important role of B lymphocytes in T1D pathogenesis [32]. However, anti-CD20 treatment does not discriminate between beneficial and pathogenic B lymphocytes, leading to a weakened immune system. The specific removal of pathogenic autoantibodies or the neutralization of pathogenic B lymphocytes would provide a specific therapeutic strategy. Anti-Id have been shown to accomplish this specific task in experimental studies [33]. To date, treatment or prevention of T1D with GAD65Ab-specific anti-Id has not been reported. However, our previous study suggested that induction of GAD65Ab-specific anti-Id prevented onset of diabetes in NOD mice [20]. Even though GAD65 levels in murine pancreatic beta cells are very low [34], GAD65 is a major autoantigen in the pathogenesis of T1D in the NOD mouse. GAD65-reactive T cells present in the NOD mouse are able to adoptively transfer T1D [35], [36], and the development of these GAD65-reactive T cells depends on the presence of GAD65-reactive B lymphocytes [37]–[39]. These findings support the hypothesis that GAD65-reactive B lymphocytes are essential in disease development.

Our results of successful prevention of T1D by a b96.11-specific anti-Id not only support our hypothesis of a protective role of anti-Id in T1D, but they may also open pathways aimed at the prevention of T1D in human.

## Materials and Methods

### Ethics Statement

Written informed consent was obtained from all participants or their legal guardian. This study was approved by the IRB of the Affiliated Hospital of Nanjing University of Traditional Chinese Medicine, China, and the IRB of the University of Washington, Seattle, USA.

The animal studies were approved by the animal care and use committee of Nanjing University of Traditional Chinese Medicine. All procedures involving animals obeyed the animal care and use regulation of the Jiangsu province.

### Patient cohorts

#### T1D patients

Sera of T1D patients (n = 10), (mean age: 21.9 years, age range: 12–29 years) were collected from 2008–2011 in Jiangsu Province Hospital of TCM, Nanjing, China.

#### T2D patients

GAD65Ab-negative sera of T2D patients (n = 22), (mean age: 59.1 years, age range: 32–86 years) were collected in Jiangsu Province Hospital of TCM, Nanjing, China. The patients were classified with T2D according to the 1997 American Diabetes Association criteria. None of these patients was diagnosed with autoimmune disease.

#### Healthy individuals

GAD65Ab-negative individuals (n = 40) (mean age: 53.3 years, age range: 23–76 years) were collected in Jiangsu Province Hospital of TCM, Nanjing, China from volunteers who received annual physical examination in the hospital agreeing to donate blood samples.

Sera from additional healthy individuals (n = 40) (age at sampling >18 years) were selected based on GAD65Ab-titer and serum volume from a cohort of healthy individuals (n = 50). The samples were collected in 2002 in Seattle, USA. This cohort consisted of non-diabetic individuals without known autoimmune disease and no family history of diabetes.

### Animals

Balb/c mice were purchased from Shanghai Slack Experimental Animal Center, Ltd (Shanghai, China) and maintained in the Experimental Animal Center of Nanjing Medical University of TCM.

Female NOD mice were purchased at 3 weeks of age (Beijing China Fukang Biological Technology Co. Ltd, Beijing, China). The mice were maintained in specific pathogen-free conditions in the animal facility under the regulation of local government.

### Antibodies used in this study

Human monoclonal antibodies b96.11 and b78 specific to GAD65 were derived from a patient with autoimmune polyendocrine syndrome type 2 [40]. B96.11 recognizes an epitope that is specifically bound by patients with T1D [41]. B78 recognizes an epitope that is specifically bound by patients with SPS [42]. N-GAD65mAb is a mouse GAD65-specific monoclonal antibody that recognizes a linear epitope located at amino acid residues 4–22 of human GAD65 [43]. The chimeric antibody hLF is specific to anthrax lethal factor and served as a control antibody. It consists of the constant region of human immunoglobulin G and the murine variable region. The mouse monoclonal antibody used as a control antibody in the NOD injection protocol was a product of one of the hybridoma generated from Balb/c mice injected with b96.11 monoclonal antibody (see below). It showed no binding to b96.11 and its specificity has not been further evaluated. Its isotype was determined as IgG1 by ELISA (Thermo Fisher Scientific China, Beijing, China).

### Generation of hybridoma cell line MAb 8E6G4

Balb/c mice (4 weeks of age) were immunized with four intraperitoneal injections, each containing 10 µg b96.11 in complete (primary injection) or incomplete (booster injections) Freund's adjuvant. Sera from immunized animals were tested for their binding to b96.11. The mouse with the highest titer was boosted with intravenous injections (both intraperitoneal and intravenously) of 10 µg of b96.11. Splenocytes were fused with SP2/0 cells by polyethylene glycol and the hybridoma cells were selected as previously described (26). Culture supernatants of confluent cells were screened at different dilutions for antibodies to b96.11 by ELISA. Cells exhibiting the highest Ab titers were cloned by limiting dilutions in 96-well microculture plates and tested for reactivity with b96.11.

### Injection of NOD mice with MAb 8E6G4

Young female NOD mice (4 weeks of age) (groups of 12) were injected intraperitoneal weekly with 50, or 100 µg antibody, or PBS. The injections continued until the animals reached 40 weeks of age or developed diabetes. All animals were monitored for the development of diabetes. Hyperglycemia was determined by weekly weighing and blood glucose level tests. Blood glucose levels were measured weekly by Abbott Medisense Optium Xceed (Abbott Laboratories S.A. Shanghai, China). Diabetes was defined by blood glucose levels of >13.9 mM for two consecutive weeks.

Upon confirmation of diabetes, the animal was killed. Degree of insulitis was established for at least 40 islets in each treatment group.

### Insulitis scoring

A minimum of 40 islets/group were scored for insulitis. Scoring was performed under double-blinded conditions. The degree of insulitis was graded according to the following: normal islet, score 1; perivascular/periductal infiltration, score 2; peri-insulitis, score 3; mild insulitis (<25% of the islet infiltrated), score 4; and severe insulitis (more than 25% of the islet infiltrated), score 5.

### Cloning of the heavy and light chain sequences

Gene fragments encoding the heavy and light chain of the antibody were amplified from 8E6G4 hybridoma by RT-PCR as described previously [13]. PCR was performed using standard procedures with Taq DNA Polymerase (Qiagen, Valencia, CA) at an annealing temperature of 60°C. The nucleotide and deduced amino acid sequence were compared against known sequences in the NCBI database.

### ELISA and competing ELISA

#### a) Detection of anti-Id specific to b96.11 by ELISA

Binding of mice serum, supernatant of hybridoma, and purified MAb 8E6G4 to b96.11 was tested by ELISA using standard procedures. Briefly, 96-well plates were coated with human monoclonal antibody b96.11 or control antibody (1 ng/µl) at 4°C overnight. Samples were added and incubated for 45 minutes at 37°C. Bound mouse IgG was detected by addition of alkaline phosphatase-conjugated anti-mouse IgG (1∶3,000) (Sigma-Aldrich China, Inc., Shanghai, China). Optical density (OD) at 405 nm was measured on Quant Microplane Spectrophotometer (Bio-Tek China, Beijing, China). For competing ELISA, 1.4 µg/ml MAb 8E6G4 was preincubated with different amounts of *in vitro* synthesized GAD65, prior to addition to the coated plates.

#### b) Detection of GAD65Ab in human sera by ELISA on immobilized MAb 8E6G4

Human sera of healthy individuals (n = 40), T1D patients (n = 10), and T2D patients (n = 22) were tested for the presence of IgG binding to MAb 8E6G4 by ELISA. 96-well plates were coated with MAb 8E6G4 (1 ng/µl) as above. After blocking with 5% non-fat milk, human sera (50 µl) was added and incubated at 37° for 30 minutes. The captured human antibody was detected by horseradish peroxidase (HRP)-conjugated goat anti-human IgG secondary antibody (1∶5,000) (Beyotime Biotechnology Ltd, Haimen, China). Optical density (OD) at 450 nm was measured on a Quant Microplane Spectrophotometer (Bio-Tek China).

### Calculation of dissociation constant

The dissociation constant of MAb 8E6G4 was calculated by non-competing ELISA. Briefly, 96-well plates were coated with different concentrations of b96.11 (1, 2, and 4 ng/µl) and incubated with serial dilutions of MAb 8E6G4 (0–16.4 nM). Bound mouse antibody was detected as described above. The Kd was calculated as the following formula: Kd = 2(nKd′-Kd)/(n-1), where n = [b96.11]′/[b96.11].

### Dot-blot

The supernatant of hybridoma 8E6G4, 50 ng of purified MAb 8E6G4, or 50 ng of bovine serum albumin were spotted onto PVD-membranes. After blocking, the membrane was incubated with 10 µg/ml b96.11 at room temperature for 90 minutes. Bound b96.11 was detected by HRP-conjugated goat anti-human IgG secondary antibody (1∶5,000) (Beyotime Biotechnology Ltd).

### Immunoprecipitation (IP) and immunoblotting

a) GAD65Ab b96.11 (10 µg) was incubated with MAb 8E6G4 (10 µg) or 1 ml of supernatant of hybridoma 8E6G4 at room temperature for 4 hours. MAb 8E6G4 was captured by protein-L coupled sepharose (80 µl) (Genscript, Hong Kong, China) at 4°C overnight with continuous rotation. The captured protein complexes were resolved by SDS-PAGE and transferred to PVD-membranes. b96.11 was detected as described under Dot blot.

b) Sera obtained from healthy individuals (1 ml) were incubated with 100 µl of MAb 8E6G4 immobilized to Sepharose beads for 10 min at 50°C followed by 30 min at 37°C and finally 10 min at room temperature. After the removal of the supernatant, the beads were washed extensively with PBS. The bound anti-Id were eluted by incubation with 0.2 M glycine, pH 10.0 and neutralized. The eluted fractions were analyzed for GAD65Ab in a RBA.

### Radioligand binding assay (RBA)

GAD65Ab were measured as described previously in a RBA [44]. Briefly, recombinant ^35^S-GAD65 was produced in an *in vitro* coupled transcription and translation system with SP6 RNA polymerase and nuclease treated rabbit reticulocyte lysate (Promega, Madison, WI, USA). The *in vitro* translated 35S-GAD65 was kept at −70°C and used within 2 weeks of preparation. Monoclonal antibodies were incubated at the indicated concentrations with ^35^S-GAD65 (25,000 of TCA precipitable radioactivity). After overnight incubation, free ^35^S-GAD65 was separated from the antibody-bound tracer by precipitation with Protein A Sepharose (Invitrogen). The immunoprecipitated radioactivity was counted on a Wallac Microbeta Liquid Scintillation Counter (Perkin Elmer Life and Analytical Sciences, Inc, Boston, MA, USA). All samples were analyzed in triplicate determinations. In the Diabetes Antibody Standardization Program (DASP) workshop 2005 the GAD65Ab analysis ranked at 80% sensitivity and 91% specificity.

### Statistical analysis

Incidence of diabetes was compared between the different NOD groups using the nonparametric log rank test. Median GAD65Ab levels were compared using the non-parametric Wilcoxon signed rank test. Significance was defined by P<0.05.
